# Synthesis, Characterization and Spectral Properties of Substituted Tetraphenylporphyrin Iron Chloride Complexes

**DOI:** 10.3390/molecules16042960

**Published:** 2011-04-06

**Authors:** Zhi-Cheng Sun, Yuan-Bin She, Yang Zhou, Xu-Feng Song, Kai Li

**Affiliations:** Institute of Green Chemistry and Fine Chemicals, Beijing University of Technology, Beijing 100124, China

**Keywords:** synthesis, porphyrin, iron porphyrin, spectra property

## Abstract

A series of substituted tetraphenylporphyrin iron chloride complexes [RTPPFe(III)Cl, R=*o*/*p*-NO_2_, *o*/*p*-Cl, H, *o*/*p*-CH_3_, *o*/*p*-OCH_3_] were synthesized by a novel universal mixed-solvent method and the spectral properties of free base porphyrins and iron porphyrin compounds were compared with each other. The experimental results showed that the one-pot mixed solvent method was superior to the two-step method in the yields, reaction time and workup of reaction mixtures for the synthesis of iron porphyrin compounds. The highest yields (28.7%–40.4%) of RTPPFe(III)Cl were obtained in the mixed solvents propionic acid, glacial acetic acid and *m*-nitrotoluene under reflux for 2 h. A detailed analysis of ultraviolet-visible (UV-vis), infrared (IR) and far-infrared (FIR) spectra suggested the transformation from free base porphyrins to iron porphyrins. The red shift of the Soret band in ultraviolet-visible spectra due to the presence of *p*-nitrophenyl substituents and the blue shift of Fe-Cl bond of TPPFeCl in far-infrared spectra were further explained by the electron transfer and molecular planarity in the porphyrin ring.

## 1. Introduction

In recent years substituted porphyrin-like complexes with conjugated macrocycles have been essential to the study of biomimetic chemistry [1,2], iatrology [3], analytical chemistry [4] and molecular electronic devices [5]. One of the most important applications of the porphyrin-like complexes is as a model for the natural enzyme peroxidase, in which the dioxygen has been activated by metalloporphyrins under mild conditions [6]. Especially, the iron porphyrin complexes are widely used as model compounds to simulate the catalytic behavior of cytochrome P450 enzymes in life processes [7]. They can be used as biomimetic catalysts to catalyze the selective oxidation of saturated hydrocarbons, aromatic hydrocarbons and their side chains with dioxygen [8,9,10]. The importance of iron porphyrin complexes in biological systems and biomimetic catalytic reactions have prompted extensive studies on the synthesis of iron porphyrin compounds [11]. Many new synthetic methods of metalloporphyrins have been developed, including the tetramerization of pyrrole [12,13,14,15,16] and the self-condensation of dipyrromethene [17,18]. Thereinto, the synthesis of substituted tetraphenylporphyrin compounds via the tetramerization of benzaldehyde and pyrrole has also been improved by using different organic oxidants [19,20], carboxylic acids [21] and solvents [22].

The tetraarylporphyrins were obtained for the first time by Rothemund [23,24]. Up to now, there exist two simple and practical methods of synthesizing porphyrin compounds. Adler and Longo firstly converted aromatic aldehyde and pyrrole to corresponding porphyrin complexes in a single refluxing carboxylic acid with air oxidation [25]. The separation of products was relatively simple using the Adler-Longo method [13], but the yields of porphyrins rarely exceeded 20%. Lindsey’s group subsequently developed another synthetic strategy to form substituted tetraphenylporphyrin compounds in CH_2_Cl_2_ solvent with BF_3_ etherate as catalyst and *p*-chloranil as oxidant [14]. The Lindsey synthetic approach was more feasible for the larger scale synthesis [26] and obtained much higher yields of substituted tetraphenylporphyrins with the addition of salts [27]. However, large diluent agents and expensive quinones were involved in the Lindsey method, which brought about the high cost of porphyrin synthesis and restricted their applications at present. Therefore, the Adler method are of the advantages of simple manipulation and ease of workup with low yields, while the Lindsey method can obviously improve the yields of porphyrins with costly and environmental impacts. Meanwhile, the synthetic methods are different for the metalloporphyrins with various structures in the porphyrin rings, which lead to the universal synthetic method impossible to be used in the porphyrin complexes with various substituents. Therefore, the study on synthetic methods for conveniently improving the yields of metalloporphyrin complexes is of great significance. In this work, a series of substituted tetraphenylporphyrin iron chloride complexes were synthesized with two different methods. Simultaneously, the structures of above porphyrin-like complexes were characterized by UV-vis, IR, FIR and elemental analysis.

## 2. Results and Discussion

### 2.1. Synthesis with One-pot and Two-step Methods

**Scheme 1:**
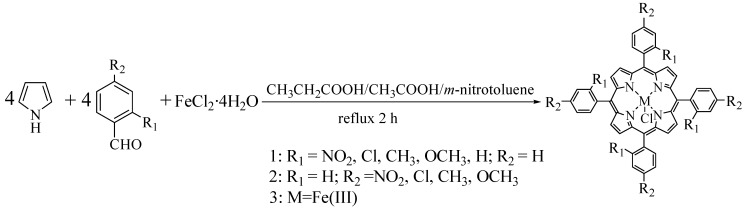
Synthesis of iron porphyrin complexes by the one-pot mixed solvent method.

**Scheme 2:**
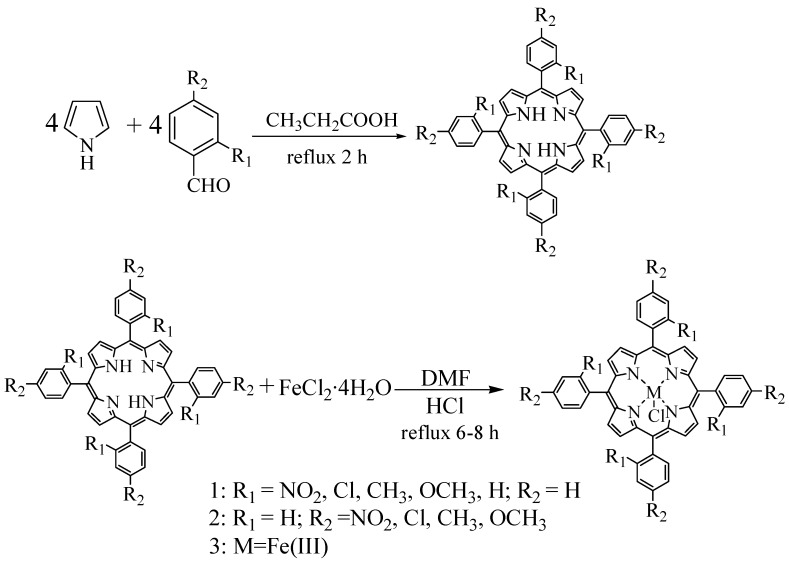
Synthesis of iron porphyrin complexes with the two-step method.

Nine substituted tetraphenylporphyrin iron chloride compounds were prepared using the one-pot mixed solvent method and the Adler two-step method. The synthetic reactions are shown in Scheme 1 and Scheme 2.

It was well known that the classic Adler two-step method of synthesizing metalloporphyrins is carried out by reacting free base porphyrins with metal salts in refluxing dimethylformamide (DMF) for a long time [28]. This method had advantages in the manipulation of synthesis and purities of products, but the yields of synthesized free base porphyrins were much lower (< 20%) and the reaction time for synthesizing metalloporphyrins was too long. Therefore, the prominent advantages of synthesizing metalloporphyrins by using one-pot mixed solvent method were that much higher yields of metalloporphyrin complexes could be obtained after shorter reaction times with simple workup of the reaction mixtures. A comparison of yields between the one-pot and two-step synthetic methods is given in Table 1.

From Table 1, it could be found that the synthetic yields of porphyrins were improved by the one-pot method, and the yields of substituted tetraphenylporphyrin iron chlorides were much higher (from 28.7% to 40.4%) than those (from 5.6% to 18.4%) in two-step method. For the synthesis of porphyrins in a single carboxylic acid (e.g. propionic acid) with air oxidation, it was difficult to turn up the appropriately corresponding conditions, especially such as polarity (dipole moment), acidity (p*K*_a_) and reaction temperature (reflux temperature), therefore, the synthetic yields of porphyrins were relatively low in the single carboxylic acid and this single-solvent method was not universal for the synthesis of different substituted porphyrins. By mixing specific carboxylic acids and *m*-nitrotoluene in some proportion, the polarity and acidity of the reaction system may be conveniently improved, further promoting the deprotonation ability of *α*-H in pyrrole and the protonation strength of C=O in the aromatic aldehyde. Simultaneously, the reflux temperature (120 ^o^C) in mixed solvents is lower than that in the single propionic acid (141 ^o^C) or DMF (153 ^o^C), which decreases the side effects caused by high reaction temperature. In addition, besides changing the polarity of the mixed solvent systems, the *m*-nitrotoluene in the mixed solvents played a role of oxidant [29], since *m*-nitrotoluene is a weak organic oxidant that can effectively promote the formation of free base porphyrins in contrast to atmospheric O_2_ as oxidant and correspondingly increased the yields of metalloporphyrins. The ratio of mixed solvents also had an important influence on the synthesis of substituted tetraphenylporphyrins. By changing the ratio of mixed solvents, the polarity of mixed solvents could be better adjusted to increase the collision frequencies of reactant molecules and accelerate the formation of porphyrins in the one-pot mixed solvent system. Moreover, the one-pot mixed solvent method was valid for different substituted benzaldehydes and the reaction time was shorter than that in the two-pot method. Therefore, the one-pot mixed solvent method was an efficient one to synthesize tetraphenylporphyrin iron compounds with different substituents.

### 2.2. Spectral Analysis of RTPPFe(III)Cl

The structures of all free base porphyrins and substituted tetraphenylporphyrin iron(III) chlorides were characterized through UV-vis and the spectral data are listed in Table 2.

By comparing the UV-vis data in Table 2, it was noticed that four absorption bands presented at the Q band and the maximum wavelength absorption (*λ*_max_) presented at the Soret band along with the formation of free base porphyrins. When the metal ion was inserted into the porphyrin ring and then coordinated with four N atoms, the iron ion located in the center of the porphyrin ring to form the iron porphyrin compounds. Then the number and intensity of the Q bands decreased and the Soret band occurred slightly red shift (e.g. *o*-ClTPP to *o*-ClTPPFeCl, from 412 nm red shift to 418 nm), which was the characteristics of iron porphyrin compounds formed. The reason might be that the structure symmetry of iron porphyrin compounds with C_4v_ point groups was improved and the energy gap decreased comparing with free base porphyrins with D_2h_ point groups. Therefore, the UV-vis spectra of iron porphyrin compounds were obviously different from those of free base porphyrins.

It also could be observed that the absorption band in the UV-vis region of iron porphyrin compounds with –NO_2_ group located at ~422 nm, which revealed the red shift compared with other iron porphyrin compounds. The reason might be that the strong electron-withdrawing –NO_2_ group decreased the electronic density of the porphyrin ring. Thus, the energy levels of π_1_ and π_2_ orbits were increased and the energy gap between HOMO and LUMO of the porphyrin ring became smaller. The π-π^*^ electron excitation of the porphyrin ring required absorbing the light of smaller energy (longer wavelength), accordingly the absorption band (Soret band) occurred red shift and located in the long wavelength region. Infrared and far-infrared spectra data of above porphyrin compounds were listed in Table 3.

As shown in Table 3, the IR absorption frequencies were different for free base porphyrins and iron porphyrin complexes with different functional groups. It was found that the N-H bond stretching and bending frequencies of free base porphyrins located at ~3,300 cm^−1^ and ~960 cm^−1^. When the iron ion was inserted into the porphyrin ring, the N-H bond vibration frequency of free base porphyrins disappeared and the characteristic functional groups of Fe-N bond formed at ~1,000 cm^−1^, which indicated the formation of iron porphyrin compounds [30]. The bands at 2,923~3,133 cm^−1^ were assigned to the C-H bond of the benzene ring and pyrrole ring. The bands at 1,494~1,682 cm^−1^ and 1,334~1,352 cm^−1^ were assigned to the C=C stretching mode and the C=N stretching vibration respectively. The bands at ~800 cm^−1^ and ~750 cm^−1^ were respectively assigned to the C-H bond bending vibration of *para*-substituted and *ortho*-substituted phenyl ring. The FIR characterization of Fe-Cl bond vibration located at 359~379 cm^−1^. The above results were in good agreement with the substituted tetraphenylporphyriniron chlorides as expected. Moreover, it could be well observed that the Fe-Cl bond vibration frequency (379 cm^−1^) of TPPFeCl was obviously higher than that in other substituted tetraphenylporphyrin iron chlorides. Generally, the vibration frequency shifted to the higher frequency region (blue shift) as the bond energy increased. Owing to the fact TPPFeCl has a MOOP structure [30] it was roughly planar and this led to better resonance effects than RTPPFeCl with a ruffling structure. Accordingly, the bond energy of Fe-Cl bond in TPPFeCl increased and the vibrational frequency greatly shifted to the higher region.

## 3. Experimental 

### 3.1. Materials and Instruments

All chemicals were obtained commercially and used as received unless otherwise noted. Pyrrole was redistilled before use. CH_2_Cl_2_ was dehydrated. Neutral Al_2_O_3_ was baked at 100 °C for 5 h. Chromatography was performed on neutral Al_2_O_3_. UV-Vis spectra were recorded in CH_2_Cl_2_ with a HITACHI U-3010 spectrophotometer. IR spectra were recorded as KBr pellets via a Nicolet AVATAR-360 spectrophotometer. FIR spectra were recorded on a Brucker FT-IR VERTEX 70 spectrometer. The data of elemental analysis were obtained with an EURO EA3000 elemental analyzer.

### 3.2. Synthesis of RTPPFe(III)Cl

#### 3.2.1. Synthesis–Adler Two-step Method

*RTPPH_2_* (R=*o/p*-NO_2_, *o/p*-Cl, H, *o/p*-CH_3_, *o/p*-OCH_3_) were synthesized by the direct condensation of pyrrole with substituted benzaldehydes according to the documented procedure [13]. The free base porphyrins were purified through a column of Al_2_O_3_ (grade III) and eluted with CH_2_Cl_2_. The solvent was then removed under vacuum and the purified porphyrin complexes were obtained in yields of less than 20%.

*o-NO_2_TPPFeCl*. *o*-NO_2_TPPH_2_ (0.2 g, 0.25 mmol) was dissolved in DMF (100 mL). The solution was heated to reflux with magnetic stirring. Upon dissolution of the *o*-NO_2_TPPH_2_, FeCl_2_·4H_2_O (0.3 g, 1.5 mmol) was added into the solution in three portions over 30 min, thin-layer chromatography (alumina, using CH_2_Cl_2_ as eluant) indicated no free base porphyrins at this point. After that, DMF (50 mL) was removed from the solution. The solution was cooled to 50–60 °C and 6 M HCl (40 mL) was added into it. The solid appeared from the solution and then was filtrated and washed with 3 M HCl until the filter cake no longer appeared green. The resulting solid was vacuum-dried to afford over 91.9% yield of *o*-NO_2_TPPFeCl. Mp > 300 °C; Anal. Calcd. for C_44_H_24_N_8_O_8_ClFe: C 59.78; H 2.74; N 12.68; Found: C 59.35, H 2.64, N 12.52; UV-vis (CH_2_Cl_2_) *λ*_max_: 422 nm (Soret band), 510 nm, 579 nm (Q band); IR (KBr) *ν*_max_: 999 cm^−1^ (*ν*_Fe-N_), 367 cm^−1^ (*ν*_Fe__-Cl_).

*o-ClTPPFeCl* was prepared using the same procedure as described for *o*-NO_2_TPPFeCl and the final solid was recrystallized from CH_2_Cl_2_ to yield 94.7% of the title compound. Mp > 300 °C; Anal. Calcd. for C_44_H_24_N_4_Cl_5_Fe: C 62.78, H 2.87, N 6.66; Found: C 62.44, H 3.16, N 7.01; UV-vis (CH_2_Cl_2_) *λ*_max_: 418 nm (Soret band), 505 nm, 576 nm (Q-band); IR (KBr) *ν*_max_: 999 cm^−1^ (*ν*_Fe-N_), 367 cm^−1^ (*ν*_Fe__-Cl_).

*o-CH_3_TPPFeCl* was prepared using the same procedure as described for *o*-NO_2_TPPFeCl and the final solid was recrystallized from CH_2_Cl_2_ in 91.5% yield. Mp > 300 °C; Anal. Calcd. for C_48_H_36_N_4_ClFe: C 59.78, H 2.64, N 12.61; Found: C 59.35, H 2.74, N 12.68; UV-vis (CH_2_Cl_2_) *λ*_max_: 416 nm (Soret band), 511 nm, 585 nm (Q-band); IR (KBr) *ν*_max_: 998 cm^−1^ (*ν*_Fe-N_), 361 cm^−1^ (*ν*_Fe__-Cl_).

*o-OCH_3_TPPFeCl* was prepared in 91.5% yield using the same procedure as described for *o*-NO_2_TPPFeCl and the final solid was recrystallized from CH_2_Cl_2_. Mp > 300 °C; Anal. Calcd. for: C_48_H_36_N_4_ClFeO_4_: C 69.96, H 4.40, N 6.80; Found: C 69.36, H 4.73, N 6.78; UV-vis (CH_2_Cl_2_) *λ*_max_: 418 nm (Soret band), 513 nm (Q-band); IR (KBr) *ν*_max_: 998 cm^−1^ (*ν*_Fe-N_), 360 cm^−1^ (*ν*_Fe__-Cl_).

*TPPFeCl* was prepared using the same procedure as described for *o*-NO_2_TPPFeCl and recrystallized from CH_2_Cl_2_/CH_3_OH to yield up to 97.4% of the target compound. Mp > 300 °C; Anal. Calcd. for C_44_H_28_N_4_ClFe: C 75.07, H 4.01, N 7.96; Found: C 74.29, H 4.07, N 7.92; UV-vis (CH_2_Cl_2_) *λ*_max_: 418 nm (Soret band), 507 nm, 572 nm (Q-band); IR (KBr) *ν*_max_: 991 cm^−1^ (*ν*_Fe-N_), 379 cm^−1^ (*ν*_Fe__-Cl_).

*p-NO_2_TPPFeCl* was prepared using the same procedure as described for *o*-NO_2_TPPFeCl and the final solid was vacuum-dried and afforded above 92.0% yield of product. Mp > 300 °C; Anal. Calcd. for C_44_H_24_N_8_O_8_ClFe: C 59.78; H 2.74; N 12.68; Found: C 59.35, H 2.74, N 12.68; UV-vis (CH_2_Cl_2_) *λ*_max_: 421 nm (Soret band), 514 nm, 584 nm (Q-band); IR (KBr) *ν*_max_: 999 cm^−1^ (*ν*_Fe-N_), 368 cm^−1^ (*ν*_Fe__-Cl_).

*p-ClTPPFeCl* was prepared using the same procedure as described for *o*-NO_2_TPPFeCl and recrystallized from CH_2_Cl_2_/CH_3_OH to yield up to 99.4% of product. Mp > 300 °C; Anal. Calcd. for C_44_H_24_N_4_Cl_5_Fe: C 62.78, H 2.87, N 7.04; Found: C 62.32, H 3.31, N 6.66; UV-vis (CH_2_Cl_2_) *λ*_max_: 417 nm (Soret band), 509 nm, 573 nm (Q-band); IR (KBr) *ν*_max_: 999 cm^−1^ (*ν*_Fe-N_), 359 cm^−1^ (*ν*_Fe__-Cl_).

*p-CH_3_TPPFeCl* was prepared in up to 90.4% yield using the same procedure as described for *o*-NO_2_TPPFeCl and the final solid was recrystallized from CH_2_Cl_2_. Mp > 300 °C; Anal. Calcd. for C_48_H_36_N_4_ClFe: C 59.78, H 2.64, N 12.61; Found: C 59.43, H 2.89, N 12.96; UV-vis (CH_2_Cl_2_) *λ*_max_: 418 nm (Soret band), 452 nm, 511 nm (Q-band); IR (KBr) *ν*_max_: 999 cm^−1^ (*ν*_Fe-N_), 360 cm^−1^ (*ν*_Fe__-Cl_).

*p-OCH_3_TPPFeCl* was prepared using the same procedure as described for *o*-NO_2_TPPFeCl. The resulting solid was washed twice with water (50 mL) and recrystallized from CH_2_Cl_2_/CH_3_OH to yield up to 95.2% of product. Mp > 300 °C; Anal. Calcd. for C_48_H_36_N_4_ClFeO_4_: C 69.96, H 4.40, N 6.80; Found: C 69.30, H 4.68, N 6.86; UV-vis (CH_2_Cl_2_): *λ*_max_: 420 nm (Soret band), 509 nm, 572 nm (Q-band); IR (KBr) *ν*_max_: 998 cm^−1^ (*ν*_Fe-N_), 359 cm^−1^ (*ν*_Fe__-Cl_).

#### 3.2.2. Synthesis–One-pot Mixed Solvent Method

*o-NO_2_TPPFeCl*: Propionic acid (50 mL), glacial acetic acid (10 mL) and *m*-nitrotoluene (10 mL) were added into a 250 mL three-neck round-bottom flask equipped with stirrer, reflux condenser and dropping funnel. The mixture was stirred under reflux for 30 min. After that, *o*-nitrobenzaldehyde (1.51 g, 10 mmol) dissolved in propionic acid (20 mL) and freshly distilled pyrrole (0.7 mL, 10 mmol) dissolved in *m*-nitrotoluene (10 mL) were simultaneously added into the flask through two dropping funnels in 15 min. After 10 min, the mixture was stopped heating and cooled to 90-100 °C, FeCl_2_·4H_2_O (2 g, 10 mmol) was added into the reaction solution and was again heated to reflux with magnetic stirring for about 50 min. When the thin-layer chromatography (alumina) indicated no free base porphyrins at this point, the reaction was stopped. The crude mixture was filtered and washed with methanol three times. The further purification by column chromatography on alumina using 100% CH_2_Cl_2_ removed the porphyrin isomers and use of acetone/ethyl acetate (1:1) as eluent gave the final products. The iron porphyrin was eluted as the second band, leading to the pure compound obtained after solvent elimination (yield up to 28.7%). Other iron porphyrin complexes (RTPPFeCl) were prepared with the same procedure as *o*-NO_2_TPPFeCl. The ratios of mixed solvents and the yields of porphyrins were listed in Table1. The spectra data of iron porphyrins were in accordance with the results of two-step method.

## 4. Conclusions

Two different methods were applied to synthesize substituted tetraphenylporphyrin iron chloride complexes and the structures of above porphyrin compounds were characterized by UV-vis, IR, FIR and elemental analysis. Compared with the two-step method, the one-pot mixed solvent method offered the advantages of higher yields, shorter reaction time and convenient workup of reaction mixtures. The yields of iron porphyrins may be improved effectively by mixing different carboxylic acids and *m*-nitrotoluene in some proportion. The highest yields (28.7%-40.4%) of RTPPFe(III)Cl were obtained by using the one-pot mixed solvent method. Additionally, the characteristics of electronic spectra and molecular vibration spectra of above porphyrin compounds were analyzed respectively. Meanwhile, the red shift of the Soret band due to the presence of *p*-nitrophenyl substituents and the blue shift of Fe-Cl bond of TPPFeCl in the vibration frequency were explained by the electron transfer and molecular planarity in the porphyrin ring.

## Figures and Tables

**Table 1:** Comparison of metalloporphyrin yields by the one-pot and two-step methods. Optimum ratios of solvents: the volume ratios of propionic acid, glacial acetic acid and *m*-nitrotoluene.

No.	Compounds	Optimum ratios of solvents (one-pot method)	Total yields (one-pot method) (%)	Total yields (two-step method) (%)
1	*o*-NO_2_TPPFeCl	70:10:20	28.7	13.1
2	*o*-ClTPPFeCl	40:20:20	31.8	9.7
3	*o*-CH_3_TPPFeCl	40:20:20	33.6	16.7
4	*o*-OCH_3_TPPFeCl	70:10:20	32.5	5.7
5	TPPFeCl	70:10:20	38.3	18.4
6	*p*-NO_2_TPPFeCl	40:20:20	30.1	5.6
7	*p*-ClTPPFeCl	40:20:20	40.4	16.7
8	*p*-CH_3_TPPFeCl	70:10:20	32.7	15.4
9	*p*-OCH_3_TPPFeCl	40:20:20	35.9	13.0

**Table 2:** UV-vis data of free base porphyrins and substituted tetraphenylporphyrin iron chlorides.

No.	Compounds	*λ*_max_ (nm)				
		Soret bands	Q bands			
1	*o*-NO_2_TPP	421	516	551	593	650
2	*o*-ClTPP	412	511	542	587	642
3	*o*-CH_3_TPP	416	512	544	589	645
4	*o*-OCH_3_TPP	417	512	545	589	643
5	TPP	417	514	538	585	620
6	*p*-NO_2_TPP	424	516	551	594	604
7	*p*-ClTPP	418	514	549	589	645
8	*p*-CH_3_TPP	419	516	551	592	647
9	*p*-OCH_3_TPP	421	518	555	593	650
10	*o*-NO_2_TPPFeCl	422		510	579	
11	*o*-ClTPPFeCl	418		505	576	
12	*o*-CH_3_TPPFeCl	416		511	585	
13	*o*-OCH_3_TPPFeCl	418		513		
14	TPPFeCl	418		507	572	
15	*p*-NO_2_TPPFeCl	421		514	584	
16	*p*-ClTPPFeCl	417		509	573	
17	*p*-CH_3_TPPFeCl	418		452	511	
18	*p*-OCH_3_TPPFeCl	420		509	572	

**Table 3:** IR/FIR data of free base porphyrins and iron porphyrin compounds.

No.	Compounds	IR (cm^−1^)						
		*ν*_N-H_ (δ_N-H_)	*ν*_=C-H_	*ν*_C=C_	*ν*_C=N_	*γ*_=C-H_	*ν*_Fe-N_	*ν*_Fe-Cl_
1	*o*-NO_2_TPP	3321 (968)	3060	1606	1349	722	—	—
2	*o*-ClTPP	3325 (967)	3056	1626	1346	750	—	—
3	*o*-CH_3_TPP	3315 (965)	3016	1599	1348	739	—	—
4	*o*-OCH_3_TPP	3322 (966)	3070	1580	1349	753	—	—
5	TPP	3309 (966)	3051	1594	1352	732	—	—
6	*p*-NO_2_TPP	3322 (967)	3055	1595	1347	800	—	—
7	*p*-ClTPP	3315 (965)	3024	1627	1349	796	—	—
8	*p*-CH_3_TPP	3317 (967)	3024	1561	1349	798	—	—
9	*p*-OCH_3_TPP	3320 (967)	2925	1596	1346	805	—	—
10	*o*-NO_2_TPPFeCl	—	2925	1607	1345	740	999	367
11	*o*-ClTPPFeCl	—	2923	1673	1334	754	999	370
12	*o*-CH_3_TPPFeCl	—	3014	1598	1332	753	998	361
13	*o*-OCH_3_TPPFeCl	—	2934	1596	1334	756	998	360
14	TPPFeCl	—	2923	1597	1340	750	991	379
15	*p*-NO_2_TPPFeCl	—	2925	1595	1346	802	999	368
16	*p*-ClTPPFeCl	—	3133	1682	1338	805	999	359
17	*p*-CH_3_TPPFeCl	—	3022	1494	1338	799	999	360
18	*p*-OCH_3_TPPFeCl	—	2923	1605	1334	810	998	359
